# Host feeding preferences of malaria vectors in an area of low malaria transmission

**DOI:** 10.1038/s41598-023-43761-z

**Published:** 2023-09-29

**Authors:** Assiyatou Gueye, El Hadji Malick Ngom, Aissatou Diagne, Baye Bado Ndoye, Mamadou Lamine Dione, Babacar Souleymane Sambe, Cheikh Sokhna, Mawlouth Diallo, Makhtar Niang, Ibrahima Dia

**Affiliations:** 1 Pole de Zoologie Medicale, Institut Pasteur de Dakar, 36 Avenue Pasteur, BP 220, Dakar, Senegal; 2 Pole Immunophysiopathologie et Maladies Infectieuses, Institut Pasteur de Dakar, 36 Avenue Pasteur, BP 220, Dakar, Senegal; 3 UMR Vecteurs Infections Tropicales et Mediterraneennes (VITROME), Campus International UCAD-IRD, Route des Peres Maristes, BP 1386, Dakar, Senegal

**Keywords:** Entomology, Ecological epidemiology

## Abstract

Studying the behaviour and trophic preferences of mosquitoes is an important step in understanding the exposure of vertebrate hosts to vector-borne diseases. In the case of human malaria, transmission increases when mosquitoes feed more on humans than on other animals. Therefore, understanding the spatio-temporal dynamics of vectors and their feeding preferences is essential for improving vector control measures. In this study, we investigated the feeding behaviour of *Anopheles* mosquitoes at two sites in the Sudanian areas of Senegal where transmission is low following the implementation of vector control measures. Blood-fed mosquitoes were collected monthly from July to November 2022 by pyrethrum spray catches in sleeping rooms of almost all houses in Dielmo and Ndiop villages, and blood meals were identified as from human, bovine, ovine, equine and chicken by ELISA. Species from the *An. gambiae* complex were identified by PCR. The types and numbers of potential domestic animal hosts were recorded in each village. The Human Blood Index (HBI) and the Manly Selection Ratio (MSR) were calculated to determine whether hosts were selected in proportion to their abundance. Spatio-temporal variation in HBI was examined using the Moran’s index. A total of 1251 endophilic *Anopheles* females were collected in 115 bedrooms, including 864 blood fed females of 6 species. *An. arabiensis* and *An. funestus* were predominant in Dielmo and Ndiop, respectively. Of the 864 blood meals tested, 853 gave a single host positive result mainly on bovine, equine, human, ovine and chicken in decreasing order in both villages. Overall, these hosts were not selected in proportion to their abundance. The human host was under-selected, highlighting a marked zoophily for the vectors. Over time and space, the HBI were low with no obvious trend, with higher and lower values observed in each of the five months at different points in each village. These results highlight the zoophilic and exophagic behaviour of malaria vectors. This behaviour is likely to be a consequence of the distribution and use of LLINs in both villages and may increase risk of residual outdoor transmission. This underlines the need to study the feeding host profile of outdoor resting populations and how domestic animals may influence malaria epidemiology in order to tailor effective malaria vector control strategies in the two villages.

## Introduction

To better understand the dynamics of malaria transmission, the identification of blood meal sources of *Anopheles* vectors is essential to assess the risk of human exposure^1^. Of the 500 described *Anopheles* species, about 60 are responsible for the majority of malaria cases^2, 3^. In most regions where they occur, *An. gambiae*, *An. arabiensis*, *An. coluzzii* and *An. funestus* are the most prevalent and most involved in malaria transmission and are therefore, considered the main vectors of malaria in sub-Saharan Africa^4–6^.

Considered for a long time as highly anthropophilic and endophilic species, several recent papers have highlighted a zoophilic tendency following the implementation of vector control strategies^7–9^. Furthermore, known for their crepuscular and nocturnal biting activity, a switch in biting behaviour has been demonstrated at times and places where humans are not protected^10, 11^. Thus, determining the trophic preferences of vectors and their human blood index are important for understanding their biting behaviour, especially in areas where additional strategies targeting residual transmission are needed to achieve pre-elimination and progress towards elimination, given the efforts and resources deployed^12^.

Although bionomic and behavioral studies can support the development of interventions or strategies targeting vectors that evade standard control measures^13^, some parameters need to be considered. First, regardless of the vector, host selection is the result of the combined effects of intrinsic preferences modulated by extrinsic factors^14^. So, even if a species has an intrinsic preference for a particular host species due to genetic factors, environmental factors and the availability or accessibility of that host will cause the mosquito to adapt to others^15^. Indeed, the blood meals of mosquito vectors such as *An. gambiae s.l.* and *An. funestus* are not uniform^10^ and can vary between houses within the same village. The proportion of a mosquito population that feeds on humans can vary depending on factors such as the location/biotope where the bites occur, the human/animal ratio, the protective measures used by the population, the spraying status of the area, the homogeneity of the mosquito in host selection or the response to an insecticide^16, 17^. Secondly, if mosquito vectors are opportunistic, this may increase their proportion and therefore the frequency of feeding on animals and humans before the latter return to their bedroom^18^. This can maintain a sufficient level of human-vector contact, one of the most important components of disease transmission used in planning and assessing the risk of vector-borne disease and the impact of vector control measures^16^. In addition, studies have shown that the presence of domestic animals near human dwellings can reduce disease prevalence and be an effective means of limiting malaria transmission^19^. In this context, it is essential to understand the key elements of vector feeding behaviour to ensure the success of strategies aimed at interrupting transmission. This will help to guide the current and future malaria prevention and elimination initiatives planned in many contexts, given the interesting results obtained using strategies that have been shown to be effective^20^. Unfortunately, the factors involved are largely unknown or insufficiently characterized. This is particularly true for the vectors involved and for environmental and demographic factors.

In Senegal, studies on trophic preferences have so far focused on the different hosts bitten, without taking into account host selection and the tendency of vectors to feed on one host species versus other available host species. Thus, all aspects related to the feeding habits of the vectors in relation to the available hosts, their relative abundance and their availability are never been taken into account.

This study aims to understand the feeding behaviour and trophic preferences of *Anopheles* vectors in two villages located in the Sudanian zone of Senegal, where decrease in malaria transmission has been observed.

## Materials and methods

### Study area

**Figure 1:**
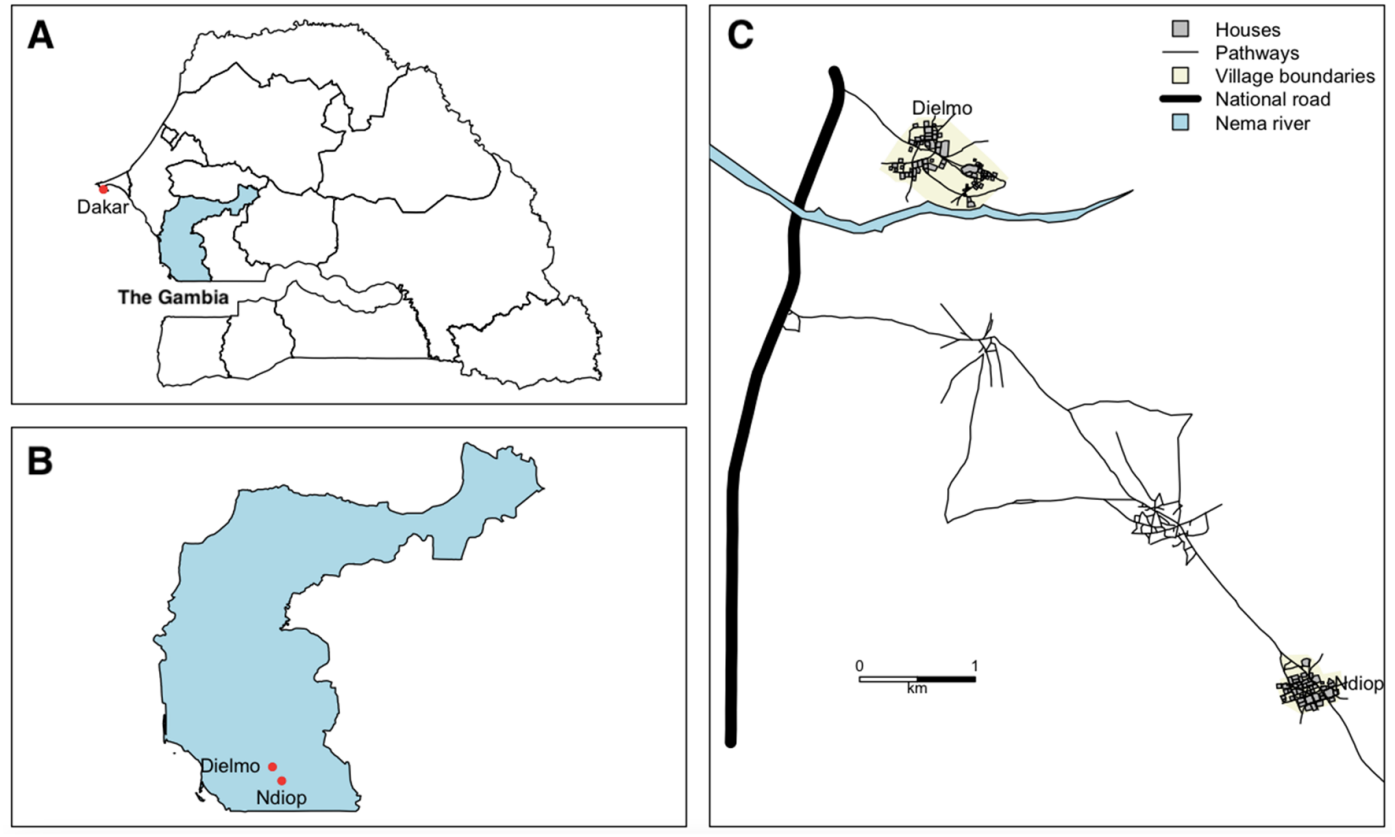
Geographical representation of the map of Senegal (**A**), the region of Fatick (**B**) and the two study villages (**C**). The Capital city of Senegal and The Gambia are shown.

The study was carried out in two localities located in the Sudanian zone of Senegal (Fig. 1): Dielmo and Ndiop, where longitudinal studies on the determinants of malaria have been carried out since 1990 and 1993, respectively^21, 22^. Since then, the epidemiology of malaria has changed to the point where elimination of the disease is envisaged^23, 24^. Unfortunately, progress has been hampered by the resurgence of malaria attacks in both villages^25, 26^. Indeed, data collected during four cross-sectional surveys conducted in the two villages prior to the malaria transmission season in June/July 2013, 2014, 2015 and 2016 identified asymptomatic infections with average parasite carriage rates of 7.7% and 9.5% in Dielmo and Ndiop, respectively^24, 26^. Such infections may act as silent reservoirs of parasites capable of sustaining low levels of residual transmission and the resurgence of clinical episodes of malaria in populations. In the villages of Dielmo and Ndiop, transmission is ensured by species of the *An. gambiae s.l.* complex and *An. funestus*^27, 28^. Around the village of Dielmo, the presence of a small river (Nema) that flows throughout the year almost ensures the permanent presence of vectors^27^. In Ndiop, transmission is seasonal because the vectors breeding sites depend on rainfall^28^. The main economic activity in both villages is agriculture, dominated by the production of groundnuts, the sale of cashew nuts, mangoes and horticulture. These two villages are mainly inhabited by the Serere and Wolof ethnic groups in Dielmo and Ndiop, respectively. The houses are of two types: traditional, with banco walls and thatched roofs, or modern, with cement brick walls and zinc roofs, with the latter being more prevalent.

### Sampling of the endophilic mosquito population and census of domestic vertebrate hosts

From July to November 2022, 115 households were systematically visited each month (69 in Dielmo and 46 in Ndiop) in the two villages for mosquito sampling. In each visited household, one bedroom was selected for sampling. In addition, geographic coordinates, household number, presence or absence of domestic animals, type of support (banco or cement walls) and number of occupants in the sampled room were recorded during a one-time interview with all household heads. A census of domestic animals was conducted by interviewing household heads during mosquito sampling in July 2022 at the beginning of the study. Mosquitoes were collected in bedrooms using the pyrethrum spray catch method between 7 and 10 am with four teams, each consisting of three individuals, for each of the two villages. Collections were made in rooms where one or more people have spent the night, using sheets spread on the floor, after spraying with pyrethroids. After each collection session, all *Anopheles* mosquitoes were identified morphologically using the identification key of Coetzee^29^ and then classified by sex and physiological status. The collected females were then individually preserved in Eppendorf tubes containing silica gel and cotton, returned to the laboratory and stored at − 20 °C for further analysis.

### Molecular identification of the *An. gambiae s.l.* species complex

Genomic DNAs from *An. gambiae s.l.* specimens were extracted from the legs or wings using the CTAB 2% method^30^. Species identification was then carried out using the techniques of Scott et al.^31^ and Fanello et al.^32^.

### Determination of the origin of blood meals

The origin of the blood meals ingested by the females was determined in the laboratory using the enzyme-linked immunosorbent assay (ELISA) technique described by Beier et al.^33^. The principle is to react on the blood ingested by the mosquito with antibodies specific for potential hosts. These antibodies are labelled by a peroxidase and, in the presence of its substrate, a coloured reaction allows the identification of the host bitten. The choice of antibodies was based on the main potential hosts present in the villages. Five different host antibodies were used: human, bovine, ovine, equine, and chicken. All blood meal sources were identified simultaneously with the five antibodies used.

### Data analysis

For each village, data related to household number, geographic coordinates, presence or absence of domestic animals, type of support (banco or cement walls) and number of occupants in the sampled room were entered into an Excel workbook, and statistical analyses were performed using R software version 4.2.0. The proportions of blood meals taken on each of the five vertebrate hosts tested were estimated by the percentage of the number of blood meals taken on each host out of the total number of blood meals identified. The human blood index (HBI) was calculated from the proportion of mosquitoes feeding on humans out of all identified blood meals. Analysis of the spatio-temporal variation of the human blood meals was performed using Moran’s index^34^ to determine autocorrelation by measuring the correlation between the households to identify the degree of clustering based on the assessed HBI (clustered, scattered, or random). Households with low or high human blood meal rates, were identified using the Getis-Ord Gi statistic*^35^. Clustered sites with high and low HBI were identified with Z scores > 1.96 and Z scores < 1.96 respectively. The selection ratio of Manly et al.^36^ was used to determine whether vectors selected human and other hosts in proportion to their relative abundance. It is estimated as the proportion of a given host among other hosts divided by the proportion of available hosts among all selectable hosts in each village. A ratio of 1 indicates that the host selection is proportional to its availability, greater than 1 if a host is selected above its proportional availability, and less than 1 if a host is selected below its proportional availability. Estimates of the selection ratio and its 95% confidence interval were calculated using the *adehabitat* package available in the R statistical software.

## Results

### Specific composition and abdominal status of female *Anopheles* mosquitoes

A total of 1251 female *Anopheles* mosquitoes were collected in the two villages (474 in Dielmo and 777 in Ndiop) between July and November 2022 (Table 1). Four morphological species were collected in Dielmo: *An. gambiae s.l.*, *An. funestus*, *An. coustani* and *An. rufipes*. *An. coustani* was not found in Ndiop. The relative abundances of the species differed (Fisher’s exact test, p < 0.05 for each village). *An. funestus* was predominant in Dielmo, whereas *An. gambiae s.l.* was the most abundant species in Ndiop. Among the species present, *An. rufipes* was the least represented. Molecular analysis of the 688 specimens of *An. gambiae s.l.* (76 and 612 from Dielmo and Ndiop, respectively) showed that the majority of the complex consisted of *An. arabiensis* with proportions of 69.7% and 67.8% respectively. In addition to *An. arabiensis*, *An. gambiae*, *An. coluzzii* and hybrids *An. gambiae*/*An. coluzzii* were collected in the two villages. The relative frequencies of the species in Dielmo were 19.7%, 5.3% and 5.3% for *An. coluzzii*, hybrids and *An. gambiae* respectively. In Ndiop the frequencies obtained were 26.6% for *An. coluzzii*, 3.9% for *An. gambiae* and 1.6% for hybrids. Most *Anopheles*, especially *An. funestus*, *An. arabiensis* and *An. coluzzii*, were collected in blood fed stage in both villages (Table 1). The feeding rates were statistically different between the two villages (χ^2^ = 10.9, ddl = 1, p = 0.001).

**Table 1:** Abdominal status of *Anopheles* species collected in Dielmo and Ndiop from July to November 2022.

Sites	Species	Fed		Gravid		Half gravid		Unfed		Total
		N	%	N	%	N	%	N	%	
Dielmo	*An. coustani*	1	50	1	50	0	0	0	0	2
	*An. funestus*	240	61.2	103	26.3	5	1.3	44	11.2	392
	*An. arabiensis*	37	69.8	11	20.8	2	3.8	3	5.6	53
	*An. coluzzii*	12	80	2	13.3	0	0	1	6.7	15
	*An. gambiae*	1	25	2	50	1	25	0	0	4
	*An. rufipes*	1	25	2	50	0	0	1	25	4
	Hybrids	3	75	1	25	0	0	0	0	4
Ndiop	*An. coustani*	0	0	0	0	0	0	0	0	0
	*An. funestus*	97	76.4	25	19.7	3	2.4	2	1.6	127
	*An. arabiensis*	298	71.8	96	23.1	7	1.7	14	3.4	415
	*An. coluzzii*	125	76.7	21	12.9	3	1.8	14	8.6	163
	*An. gambiae*	17	70.8	6	25	0	0	1	4.2	24
	*An. rufipes*	27	71.1	3	7.9	0	0	8	21.1	38
	Hybrids	5	50	4	40	1	10	0	0	10

N: number.

### Composition and representativeness of potential vertebrate hosts

**Figure 2:**
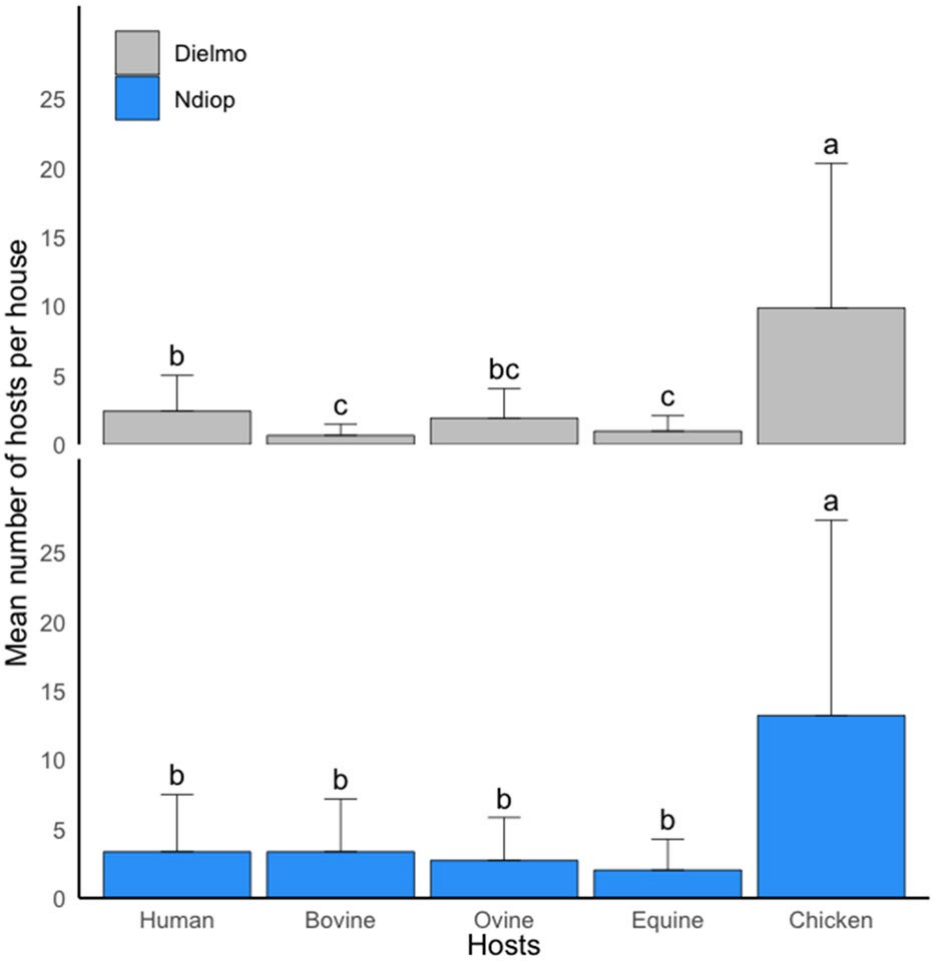
Variations in the relative abundance of the main hosts in Dielmo and Ndiop. For the five hosts, means with different letters are significantly different.

A total of 2807 animals were recorded in the two villages (1469 in Dielmo and 1338 in Ndiop). In Dielmo, 16 potential hosts were listed, compared to 12 in Ndiop, including humans. In both villages, chickens were the most common hosts, followed by caprine and human hosts. The other most common hosts were ovine in Dielmo and bovine in Ndiop. When considering the most representative hosts (bovine, ovine, chicken and equine), a significant difference was observed between the proportions of chickens (p < 0.001) compared to all other hosts in both villages (Fig. 2). Analysis of the average number of hosts per house showed that, irrespective of the site, the average number of chickens per house was much higher than the other hosts. While the average number of humans was comparable to the other hosts in Ndiop, it was different in Dielmo, except for ovine.

Due to the predominance of humans, bovine, ovine, equine and chickens, the following analyses are carried out on these five vertebrate hosts.

### Trophic preferences of vectors

A total of 864 blood meals were tested by ELISA for host identification, with 295 from Dielmo and 569 from Ndiop. Out of these blood meals, 853 were successfully identified. Most of the blood meals were taken from only one of the five vertebrate hosts tested (human or animal). The percentage of unidentified meals was 1% in Dielmo and 1.4% in Ndiop. In contrast, 2% of blood meals in Dielmo and 14.3% in Ndiop were taken from two vertebrate host species. Mixed blood meals were only observed for *An. funestus* in Dielmo, but were more common in Ndiop, primarily involving animal/animal combinations for *An. funestus*, *An. arabiensis*, *An. coluzzii*, *An. gambiae* and *An. rufipes*. No blood meal was taken from chicken in Ndiop, while only one meal was taken by *An. funestus* in Dielmo. For *An. gambiae*, no feeding on humans was observed in both villages. Only one blood meal from a horse was observed in Dielmo. Blood meals on all other hosts were identified in *An. arabiensis* and *An. coluzzii* in both villages. As with *An. arabiensis* and *An. coluzzii* in both villages and *An. gambiae* in Ndiop, blood meals were preferably taken from bovine. Among the eight *An. gambiae*/*An. coluzzii* hybrids observed in the two villages, they fed on humans, bovine and equine hosts (Table 2). *An. rufipes* and *An. coustani* did not feed on humans. In Dielmo, *An. rufipes* primarily fed on bovine hosts, while *An. coustani* fed on ovine hosts. In Ndiop, *An. rufipes* mainly fed on bovine and equine hosts (Table 2).

**Table 2:** Origin of *Anopheles* blood meals in Dielmo and Ndiop from July to November 2022.

Sites	Species	Tested	Single blood meals					Mixed blood meals		Negative
			Human	Bovine	Ovine	Chicken	Equine	AA	HA	
Dielmo	*An. funestus*	240	48 (20)	87 (36.3)	25 (10.4)	1 (0.4)	71 (29.6)	4 (1.7)	2 (0.8)	2 (0.8)
	*An. arabiensis*	37	8 (21.6)	14 (37.9)	3 (8.1)	0 (0)	11 (29.7)	0 (0)	0 (0)	1 (2.7)
	*An. coluzzii*	12	4 (33.4)	6 (50)	1 (8.3)	0 (0)	1 (8.3)	0 (0)	0 (0)	0 (0)
	*An. gambiae*	1	0 (0)	0 (0)	0 (0)	0 (0)	1 (100)	0 (0)	0 (0)	0 (0)
	*An. rufipes*	1	0 (0)	1 (100)	0 (0)	0 (0)	0 (0)	0 (0)	0 (0)	0 (0)
	*An. coustani*	1	0 (0)	0 (0)	1 (100)	0 (0)	0 (0)	0 (0)	0 (0)	0 (0)
	Hybrids	3	1 (33.3)	1 (33.3)	0 (0)	0 (0)	1 (33.3)	0 (0)	0 (0)	0 (0)
Ndiop	*An. funestus*	97	8 (8.2)	47 (48.5)	5 (5.2)	0 (0)	27 (27.8)	3 (3.1)	6 (6.2)	1 (1)
	*An. arabiensis*	298	15 (5)	141 (47.3)	7 (2.3)	0 (0)	84 (28.2)	43 (14.5)	6 (2)	2 (0.7)
	*An. coluzzii*	125	10 (8)	65 (52)	4 (3.2)	0 (0)	25 (20)	18 (14.4)	1 (0.8)	2 (1.6)
	*An. gambiae*	17	0 (0)	11 (64.7)	2 (11.8)	0 (0)	2 (11.8)	0 (0)	1 (5.9)	1 (5.9)
	*An. rufipes*	27	0 (0)	19 (70.4)	1 (3.7)	0 (0)	3 (11.1)	2 (7.4)	0 (0)	2 (7.4)
	*An. coustani*	0	0 (0)	0 (0)	0 (0)	0 (0)	0 (0)	0 (0)	0 (0)	0 (0)
	Hybrids	5	1(20)	3(60)	0 (0)	0 (0)	1(20)	0 (0)	0 (0)	0 (0)

### Spatio-temporal variations of HBI

**Figure 3:**
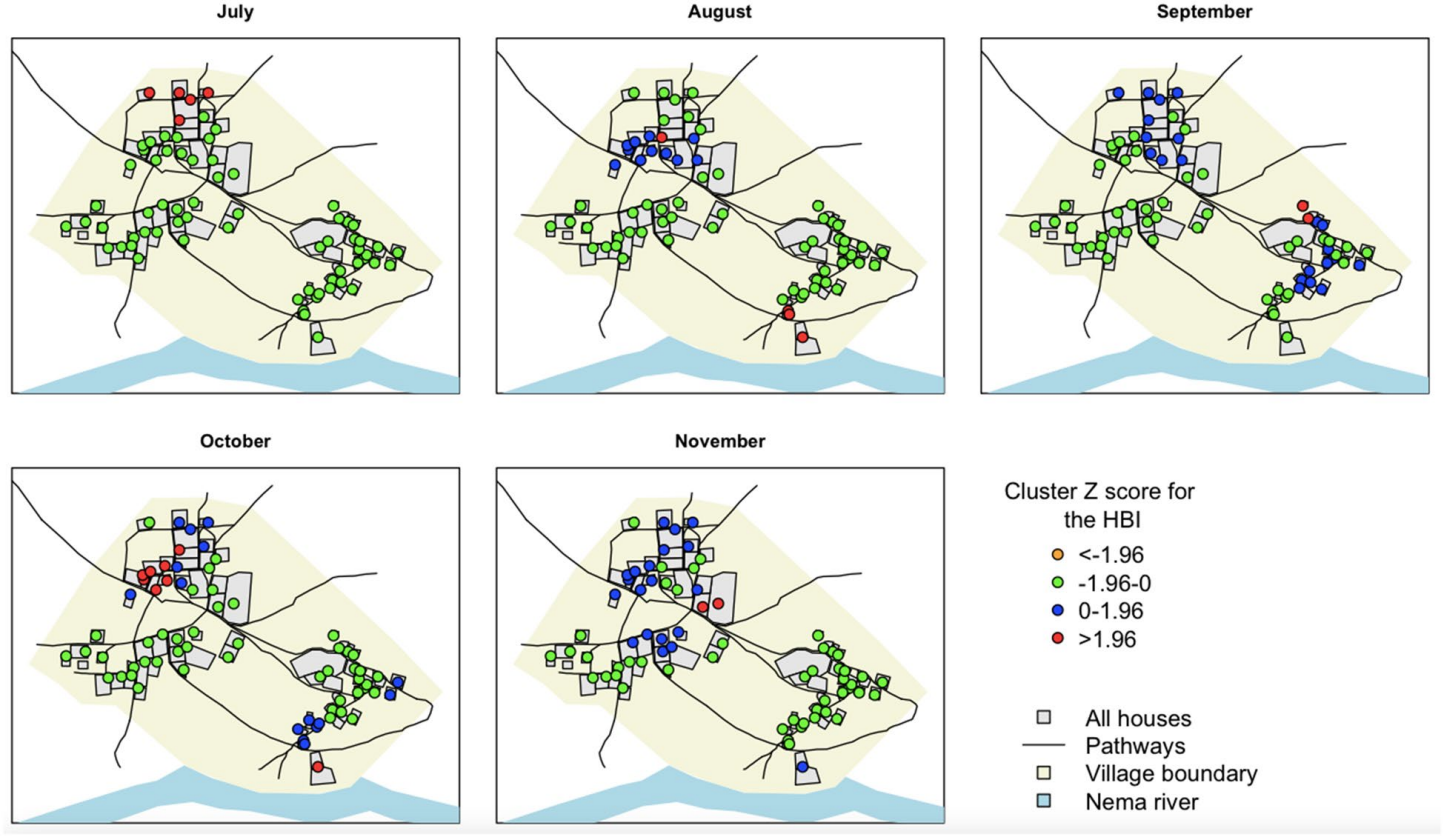
Spatial and temporal distribution of HBI of malaria vectors in Dielmo. Significance levels are analysed using Getis-Ord Gi* statistics. Z scores < 0 indicate a clustering tendency of low HBI and Z scores > 0 indicate a clustering tendency of high HBI. Significant Z scores (p < 0.05) are shown in red.

**Figure 4:**
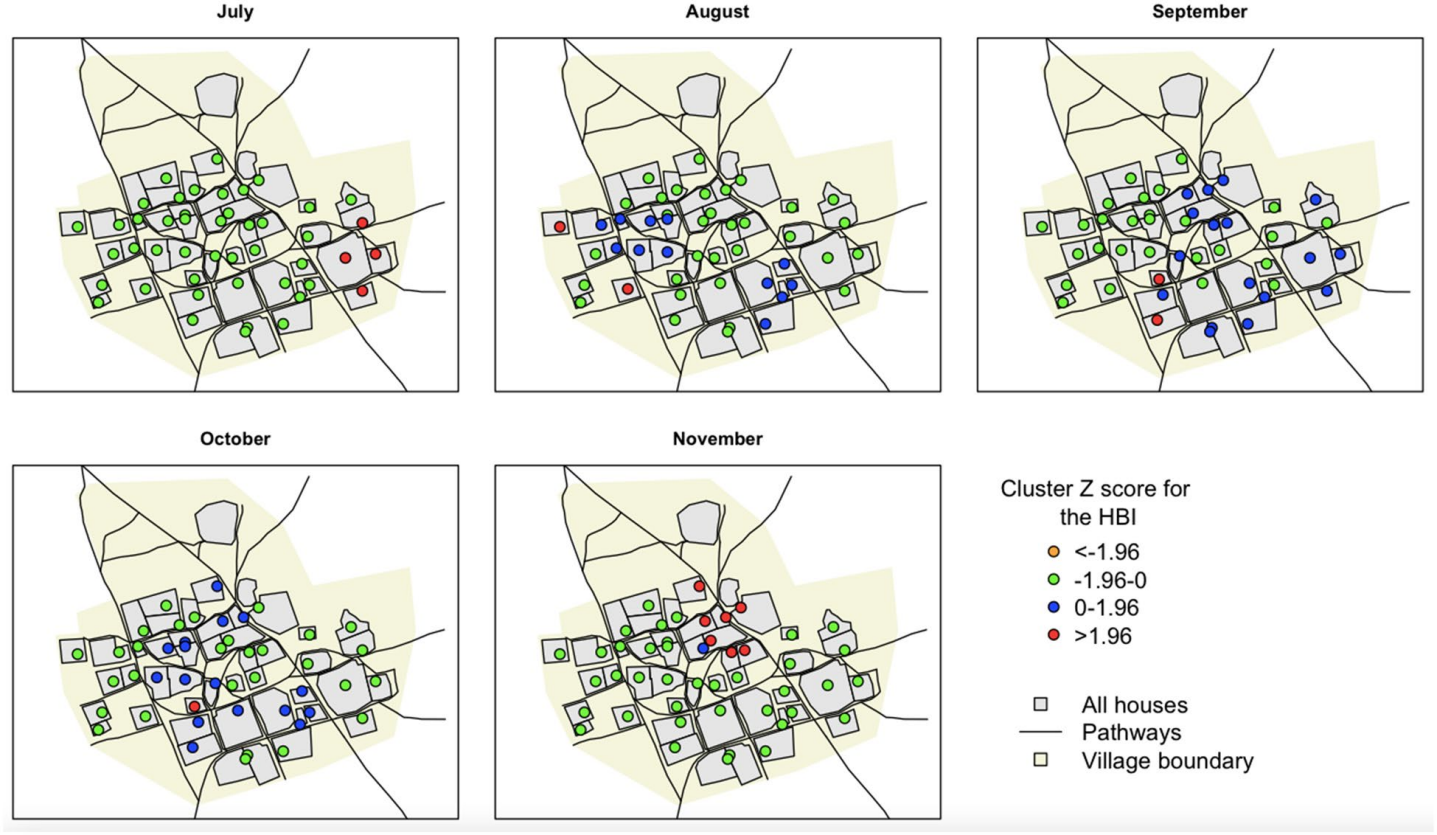
Spatial and temporal distribution of HBI of malaria vectors in Ndiop. Significance levels are analysed using Getis-Ord Gi* statistics. Z scores < 0 indicate a clustering tendency of low HBI and Z scores > 0 indicate a clustering tendency of high HBI. Significant Z scores (p < 0.05) are shown in red.

The spatio-temporal analysis of the overall HBI (all species) using spatial autocorrelation showed a negative spatial autocorrelation of the HBI in Dielmo for all months (Fig. 3, Table 3). The Z scores resulting from the hotspot analysis indicate the presence of 5, 4, 2, 6, and 2 households with significantly different Z scores from the other households in July, August, September, October and November, respectively. It’s worth noting that the majority of these households had significantly low Z scores (Fig. 3). Among these households, only one was of the traditional type, while the others were of modern type. Similarly, a negative spatial autocorrelation of the HBI was observed in Ndiop (Fig. 4, Table 3). The Z score analysis showed that in November, 7 households had significantly different values. In the other months, 4, 2, 2 and 1 households were observed in July, August, September and October, respectively.

**Table 3:** Moran’s I spatial autocorrelation indices for the HBI in Dielmo and Ndiop from July to November 2022.

Sites	Months	Moran's I	Z scores	P value
Dielmo	July	− 0.026	1.016	0.99
	August	− 0.030	1.939	0.60
	September	− 0.029	2.084	0.59
	October	− 0.005	3.479	0.44
	November	− 0.066	3.617	0.76
Ndiop	July	− 0.026	1.646	0.66
	August	− 0.047	2.779	0.62
	September	− 0.035	3.209	0.55
	October	− 0.073	2.844	0.70
	November	− 0.035	1.913	0.93

### Host selection ratio

**Figure 5:**
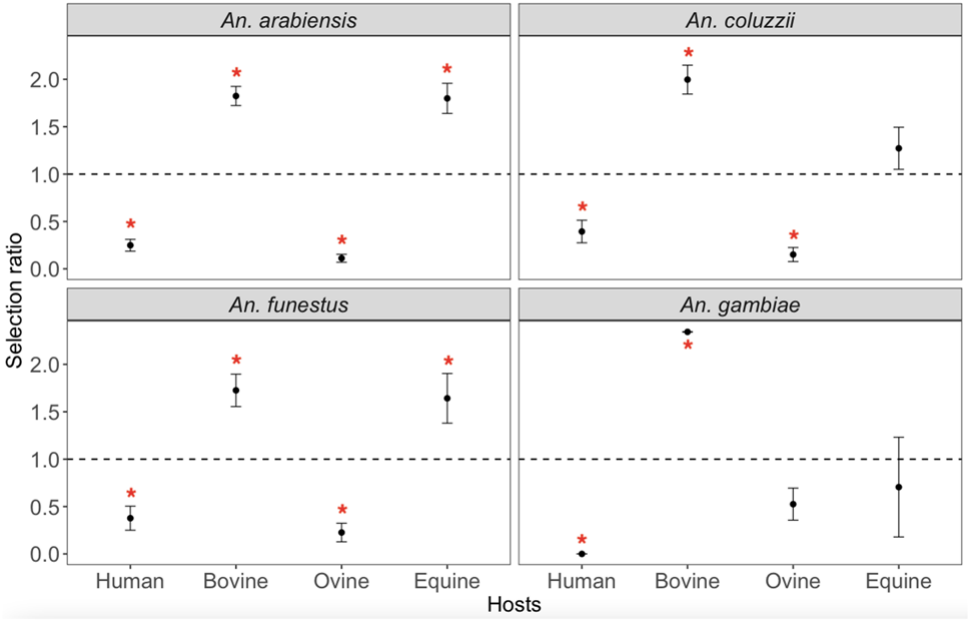
Graphs showing the Manly host selection ratio with 95% confidence interval bars for human, bovine, ovine and equine in Ndiop from July to November 2022. The dashed horizontal line marks the Manly ratio value of 1. The red asterisks indicate Manly ratios significantly greater than 1 (host species over-selection) or less than 1 (under-selection).

**Figure 6:**
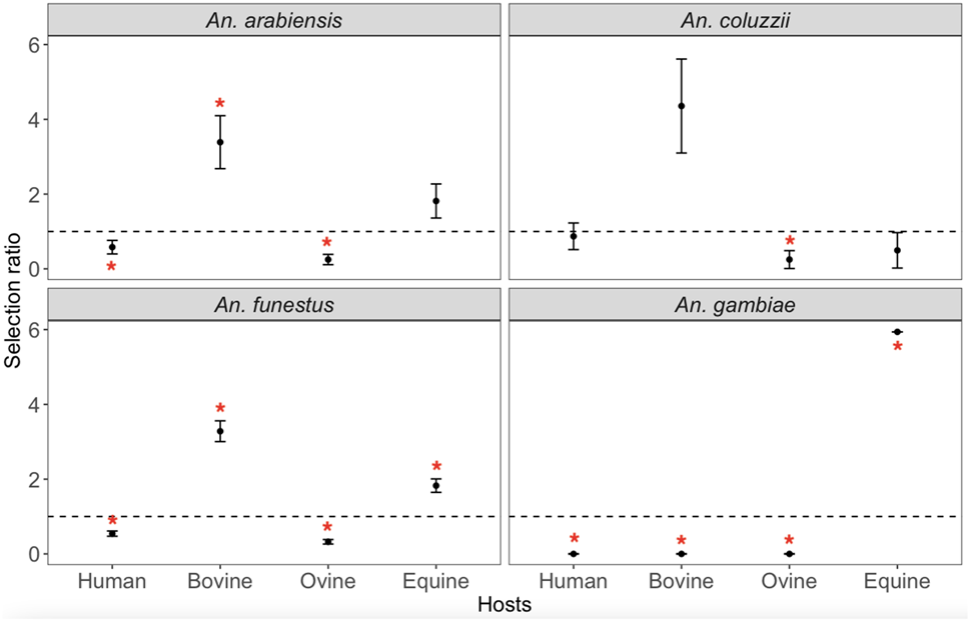
Graphs showing the Manly host selection ratio with 95% confidence interval bars for human, bovine, ovine and equine in Dielmo from July to November 2022. The dashed horizontal line marks the Manly ratio value of 1. The red asterisks Manly ratios significantly greater than 1 (host species over-selection) or less than 1 (under-selection).

The Manly selection ratio analysis showed a similar host selection profile between *An. arabiensis* and *An. funestus,* which are the the predominant species in Ndiop village. These two species were significantly under-selected for humans and ovine hosts, in contrast to bovine and equine hosts, which were over-selected (Fig. 5). *An. coluzzii* exhibited a similar profile, except that the over-selection for equine hosts was not statistically significant. In the case of *An. gambiae*, human and bovine were significantly under- and over-selected respectively, while ovine and equine were under-selected, but not significantly. In Dielmo, *An. arabiensis* and *An. funestus* significantly under-selected human and ovine hosts, while over-selecting bovine and equine hosts. However, it is important to note that the over-selection of equine hosts by *An. arabiensis* was not statistically significant. *An. coluzzii* exhibited under-selection for human, ovine and equine hosts, with only bovine hosts not showing a statistically significant over-selection (Fig. 6). As for *An. gambiae*, only one blood meal was recorded from equine hosts.

## Discussion

The identification of blood meal sources of *Anopheles* vectors is important in epidemiological studies of malaria since, for instance to understand changes in mosquito behaviour in response to long-term control efforts^37^. Seven *Anopheles* species were collected in this study, with *An. funestus* and *An. arabiensis* being the most abundant, consistent with previous findings in the area by Doucoure et al.^10^, Thiaw et al.^38^ in Dielmo and by Sokhna et al.^39^ in Ndiop.

In both villages, the relatively higher number of resting blood fed females collected from human dwellings could be related to their endophilic behaviour. These findings support studies by Fornadel and Norris^40^, which also demonstrated endophilic behaviour of *Anopheles* malaria vectors. Recent studies by Mbewe et al.^41^ in Malawi also similarly demonstrated the endophilic behaviour of *An. gambiae s.l.* and *An. funestus*. Our analysis of the blood meal sources showed that single blood meals from either humans or animals were more common than mixed blood meals from two different vertebrate hosts. Similar results were obtained by Ngom et al.^1^ in other sites in Senegal, while the results of a study conducted by Konate et al.^42^ in Dielmo showed the predominance of mixed blood meals. The majority of the identified blood meals were of animal origin, with only 11.2% originating from humans. Among the identified potential animal hosts, bovine was the most frequently bitten host (16 in Dielmo and 12 in Ndiop). Our findings are consistent with those of Massebo et al.^43^ in Ethiopia and Finney et al.^44^ in Madagascar, both of which demonstrated zoophagic feeding behaviour in *Anopheles* mosquitoes. These observations suggest that the majority of *Anopheles* collected indoors during the present study took their blood meal outdoors and then entrered the rooms to complete their trophogonic cycle. This exophagic and endophilic behaviour of *Anopheles* mosquitoes observed during our study, has been previously described by Sougoufara et al.^45^ in Dielmo. Thus, among the other types of blood meals identified, including those from humans, a large proportion was likely taken outdoors. This outdoor biting behaviour therefore poses a significant threat to elimination efforts, as it can sustain malaria transmission even in areas with high control coverage measures^46^. In both villages, the zoophagic behaviour of the vectors could be attributed to the good coverage and use of bed nets and other protective measures. This zoophagic behaviour contrasts with the findings of Konate et al.^47^, who showed that the majority of blood meals of *An. gambiae* and *An. funestus* were from humans, indicating a highly anthropophilic nature of these species. However, the current behaviour of these species has changed considerably as a result of the implementation of the many control strategies^48^. Indeed, the studies by Wotodjo et al.^25, 49^ and Doucoure et al.^10^ showed that the number of malaria cases decreased after the introduction of long-lasting insecticidal-treated nets (LLINs). The results obtained by Sougoufara et al.^45^ also showed that the use of LLINs correlated with a significant reduction in mosquito biting rates, thereby reducing disease transmission.

Due to the inaccessibility of humans, vectors have become opportunistic and feed mainly on animals^50^. This phenomenon is interesting because in both villages, human hosts are not more prevalent than other animal hosts (especially bovine, ovine, equine and chicken). This is confirmed by the results of the selection ratio study, which showed variations between different species within the same village. Regardless of location and species, bovine was significantly over-selected. *An. arabiensis*, *An. coluzzii* and *An. funestus* can be considered opportunistic, as they over-selected bovine and, to a lesser extent, equine hosts. A number of factors and processes influence mosquito host selection, such as host availability, density and accessibility^18^. Therefore, keeping people and animals together in the same house can be considered as an effective strategy for malaria control. Indeed, when mosquitoes cannot access humans, they may resort to animals to ensure their survival^41^. In addition, studies have shown that a high density of cattle around a household still provides some protection to humans, especially against mosquitoes that enter houses to feed^51^.

No human blood meals were observed in *An. rufipes* and *An. coustani*. This strictly zoophilic behaviour of *An. rufipes* observed in our study was previously reported by Konate et al.^42^ despite the presence of human hosts. These results suggest that in Senegal, *An. rufipes* may not be involved in malaria transmission, in contrast to studies conducted in Cameroon by Tabue et al.^52^, and a more recent study in southeastern Zambia by Saili et al.^46^, both of which reported the involvement of this species as a vector of human *Plasmodium*. For mixed blood meals, the most common combination was bovine and equine.

Although chickens were the most common potential domestic hosts in both villages, no blood meals were taken from this host in Ndiop, and only one blood meal was recorded from *An. funestus* in Dielmo. These results suggest that the vectors did not feed on chickens, despite their high density in both villages. Such observations have been made previously in this area by Konate et al.^48^. The wider distribution of bovine blood meals can be attributed to the abundance of these hosts. In fact, in nearly all the households visited, bovines are kept inside the houses. Thus, due to their number, size and behaviour, bovine can be considered as the most accessible hosts for mosquitoes. The results of the spatio-temporal analysis of the HBI using spatial autocorrelation revealed that regardless of the month, there was a negative spatial autocorrelation in the HBI observed in both villages. However, some households exhibited significantly higher Z scores, indicating higher frequencies of the HBI than others. This highlights the unequal vulnerability of individuals to malaria mosquito bites, despite control measures^41^. Factors that may explain this situation include the tendency of some people to spend more time outdoors or use bed nets irregularly^26^. This could be considered a risk factor for malaria transmission^46^. Additionally, the protective effect of LLINs can be reduced when individuals receive mosquito bites outside their bedrooms.

## Conclusions

Determining the blood sources of female *Anopheles* mosquitoes is essential for guiding the design of new vector control strategies. This study demonstrated the exophagic, zoophagic and opportunistic behaviors of the main malaria vectors *An. gambiae s.l.* and *An. funestus*, as well as the strictly zoophagic behaviour of *An. rufipes*, despite the presence of human hosts. The detection of human blood in both vector species suggests the potential for malaria transmission in these two communities. Furthermore, the study indicated that the presence of domestic animals near human settlements, combined with the use of LLINs, could be integrated into malaria vector control strategies. Nevertheless, further additional research is required to investigate the relationship between the observed zoophagy and potential infection of vectors by *Plasmodium* parasites. This will provide a more comprehensive understanding of residual transmission. Furthermore, investigating the feeding host profile of outdoor resting populations and studying how the presence of domestic animals may influence malaria epidemiology is necessary to tailor effective malaria control strategies.

## Data Availability

All data generated or analyzed during this study are included in this published article.
